# On the Germinal Matter of the Blood, with Remarks upon the Formation of Fibrin

**Published:** 1866-02

**Authors:** Lionel S. Beale


On the Germinal Matter of the Blood, with Remarks upon the Formation of Fibrin.—By Lionel S. Beale, M. B.—Applying his general views upon "germinal matter" and "formed material" to the blood, Dr. Beale believes that the white blood-corpuscle consists of the former, the red corpuscle of the latter, and that in red corpuscles possessing nuclei, the nucleus represents the germinal matter. In regard to a cell-wall, Dr. Beale urges some striking observations disproving its existence. Taking, for illustration, the red blood-corpuscle of the frog, the outer part which cannot be colored by carmine, is "formed material" which was once in the state of "germinal matter," and was capable of producing matter like itself, and of being resolved into colored formed material. But this formed material once produced cannot form matter like itself, although it can be changed, dissolved, and converted into other substances. It is the seat of physical and chemical actions alone. Vital changes are restricted to the germinal matter. In the nutrition of the blood-corpuscle, pabulum passes through the outer colored portion into the germinal matter, where it acquires the same vital powers which the germinal matter already existing possesses. When blood becomes stationary the white corpuscles and the nuclei of the red increase in size and subdivide. In such circumstances, "granular corpuscles" may be developed from the white or from young red corpuscles. In adult, mammal's blood are certain corpuscles, and granular in appearance, colorless, and much smaller than the ordinary red corpuscles; so small, in fact, that they can only be seen by powers magnifying upwards of 1000 diameters. These corpuscles, the author believes, to consist of germinal matter which has just commenced to undergo conversion into red formed material. The red corpuscles vary in size much more than is commonly supposed, and they differ very much in transparency and refractive power, some being only just visible in consequence of the extreme transparency of the material of which they are composed.

In the second paper quoted above, Dr. Beale advances some very remarkable views. He believes in the existence of very minute particles of germinal matter (less than the 1-4000th of an inch in diameter) in the blood. These masses of germinal matter multiply by a protrusion taking place from the surface, and final separation into two separate masses. Nuclei may arise in these masses. In inflammation of the vessels of the frog's foot, the increase of white corpuscles is not alone dependent on arrest of the circulation, but the corpuscles actually multiply in the clot. In regard to the development of cells in exudations, the author advances the view that "exudations," although clear fluids, really contain a multitude of extremely minute particles of living matter, which are intimately related to the white blood-corpuscles and that these grow and become one source of small "granular cells" or corpuscles. Further, living or germinal matter may retain its vitality, so that particles may be formed in one organism and be carried to another where they may grow. It seems to the author probable "that many contagious diseases are due not to the propagation and transference of vegetable organisms, but to small particles of living animal matter which have descended from the germinal matter of one organism, and have been transferred to another." Finally, in reference to the formation of fibrin, Dr. Beale holds that it is "the result of vital process," that it is "formed material," and "that living germinal matter becomes fibrin."—*Edinburgh Med. Jour. and Jour. of Micros. Science*, vol. iv., 1864.

